# A Hierarchical Deep Fusion Framework for Egocentric Activity Recognition using a Wearable Hybrid Sensor System

**DOI:** 10.3390/s19030546

**Published:** 2019-01-28

**Authors:** Haibin Yu, Guoxiong Pan, Mian Pan, Chong Li, Wenyan Jia, Li Zhang, Mingui Sun

**Affiliations:** 1 College of Electronics and Information, Hangzhou Dianzi University, Hangzhou 310018, China; shoreyhb@hdu.edu.cn (H.Y.); pgx@hdu.edu.cn (G.P.); ai@hdu.edu.cn (M.P.); 172040046@hdu.edu.cn (C.L.); 2 Department of Electrical and Computer Engineering, University of Pittsburgh, PA 15261, USA; wej6@pitt.edu; 3 School of Computer Science and Technology, Hangzhou Dianzi University, Hangzhou 310018, China; zhangli@hdu.edu.cn; 4 Department of Neurological Surgery, University of Pittsburgh, PA 15213, USA

**Keywords:** deep learning, egocentric activity recognition, hierarchical fusion framework, wearable sensor system

## Abstract

Recently, egocentric activity recognition has attracted considerable attention in the pattern recognition and artificial intelligence communities because of its wide applicability in medical care, smart homes, and security monitoring. In this study, we developed and implemented a deep-learning-based hierarchical fusion framework for the recognition of egocentric activities of daily living (ADLs) in a wearable hybrid sensor system comprising motion sensors and cameras. Long short-term memory (LSTM) and a convolutional neural network are used to perform egocentric ADL recognition based on motion sensor data and photo streaming in different layers, respectively. The motion sensor data are used solely for activity classification according to motion state, while the photo stream is used for further specific activity recognition in the motion state groups. Thus, both motion sensor data and photo stream work in their most suitable classification mode to significantly reduce the negative influence of sensor differences on the fusion results. Experimental results show that the proposed method not only is more accurate than the existing direct fusion method (by up to 6%) but also avoids the time-consuming computation of optical flow in the existing method, which makes the proposed algorithm less complex and more suitable for practical application.

## 1. Introduction

In recent years, wearable smart devices have advanced rapidly, and various smart wearable devices, such as Google Glass, Microsoft SenseCam, and Apple Watch have become very popular. With the help of a variety of sensors integrated in wearable devices, such as cameras, inertial measurement units (IMUs), and global positioning system (GPS), the wearer’s state data (called egocentric or first-person data) can be automatically collected and recorded over extended periods. Through analysis of these egocentric data, real-time monitoring and state evaluation of the wearer can be realized, which results in egocentric data having wide application prospects in medical care [1,2], smart homes and offices [3], security monitoring [4], and other fields. As egocentric activity recognition is the basis of subsequent activity analysis, it has an important research value and has attracted high interests in the fields of human activity recognition and artificial intelligence [5,6].

For egocentric activity recognition, traditional methods use a wearable sensor, such as an IMU, to collect motion data and then determine the activity using a machine learning-based classifier [7]. Because the IMU-produced motion data lack environmental contexts, traditional methods are sensitive to the posture of the body and the location of the sensor, which limit the recognition performance. Currently, these methods can only produce reasonable recognition results for activities with obvious differences in motion states, such as sitting, standing, walking, and climbing stairs, under the condition that the motion sensors are placed at appropriate locations of the body, which are often inconvenient or obtrusive for the device wearer. Compared with motion sensor data, video/images contain more contextual and environmental information. Consequently, vision-based activity recognition using a wearable camera has become a major focus of research in the field of egocentric activity recognition [8,9].

Recently, with the widespread study of artificial intelligence, deep learning-based methods have been developed to recognize egocentric activities [10,11,12]. Generally, these methods can be classified into two categories according to the data recording mode utilized by the wearable camera: video stream-based methods and photo stream-based methods [13]. In the first category, the video stream acquired using a short-time acquisition device, such as Google Glass, with a high shooting frame rate (above 30 frames per second/fps). Due to the availability of large amounts of correlated image data in a relatively short period of time, it is convenient to obtain the wearer’s motion information by using motion estimation methods. Thus, video stream-based methods can be used primarily to recognize transient actions or activities that are short-lived, such as “taking a medical pill” and “dipping a piece of fry in ketchup.” Conversely, photo streams are usually acquired at a low frame rate (2–3 frames per minute/fpm) using a long-time acquisition device such as a life logger. Because the wearer’s motion information cannot be estimated adequately from the low-frame-rate photo streams, this category of methods is mainly used to recognize some long-lasting high-level activities, such as most activities of daily living (ADLs) (e.g., “eating dinner” and “watching TV”).

Although the deep learning-based vision egocentric activity recognition approach has made significant progress, this approach is still subject to many conditions, such as the location of the wearable camera, imaging quality, lighting condition, and occlusion. In practical applications, no single sensor can handle all situations, and a common practice for avoiding the risk of misrecognition is to use multiple sensors. Recently, researchers have begun to fuse vision and other types of sensor data in egocentric activity recognition [2,14]. For example, an 80.5% average accuracy of 20 distinct ADLs was achieved whereas the average individual accuracies of video and sensor data only 75% and 49.5%, respectively. Although the accuracy of the activity recognition after fusion has been improved, in the hybrid sensor system, all sensors work simultaneously and equally to distinguish all activities. As different types of sensors have different characteristics and are suitable for different activity classification modes, when they work in the same classification mode, the recognition effects of some activities may be very different, which has a negative impact on the fusion results.

In order to solve the problems in existing methods, we propose a deep-learning based hierarchical fusion framework for ADL recognition in our self-developed hybrid sensor system. The main contributions of this paper are as follows:

(1) A two-layer hierarchical framework is proposed. These layers are established by mapping or making correspondence between the ADLs to be recognized and their common motion states. With this mapping or correspondence, the target ADLs to be recognized can be easily grouped in the motion state layer using motion sensor data only so that the ADL recognition can be performed in the group with a significantly reduced number of candidate activities.
(2) A novel method to use hybrid data for egocentric ADL recognition is proposed. The motion sensor data are used only to distinguish motion states of ADLs instead of distinguishing all the ADLs to be recognized. This approach enables both motion sensor data and photo streams to be used in their most suitable classification modes. This strategy effectively eliminates the negative influence of sensor differences on data fusion results.
(3) A multimodal hybrid database is created consisting of synchronized egocentric IMU sensor data and a photo stream acquired by eButton, a self-developed wearable device [15]. This database can be used to evaluate the egocentric activity recognition methods based on multisensor fusion.

The remainder of this paper is organized as follows. Related work is briefly summarized in Section 2. The proposed method is described in detail in Section 3. Experimental results are presented in Section 4. We conclude this paper and suggest future work in Section 5.

## 2. Related Work

At present, egocentric activity recognition methods reported in the literature can mainly be divided into three categories according to wearable sensor types [16]: single motion sensor-based, single camera-based, and hybrid sensor system-based methods.

### 2.1. Single Motion Sensor-Based Egocentric Activity Recognition

Motion sensors for human activity recognition mainly include accelerometers, gyroscopes, and magnetic sensors (A/M/G). These three sensors are commonly integrated in a single chip called the inertial measurement unit (IMU), which is commonly found in a variety of wearable devices. Currently, the IMU-produced motion data are the most widely used data form for egocentric activity recognition [7,17,18,19,20]. In many systems, the sampling rate of a motion sensor is selected to be very high so that the motion sensor data can be regarded as a continuous time series. From these data, motion sensor-based activity recognition methods usually extract a set of statistical features, such as the mean, variance, correlation, and amplitude area, within an overlapping or non-overlapping time sliding window. Activity recognition is then performed by a machine learning algorithm, such as a decision tree, Bayes’ law, or a support vector machine. Recently, with the progress of deep learning study, long short-term memory (LSTM) based on a recurrent neural network has been widely used in the field of time domain signal processing. Several reports have also used LSTM for egocentric activity recognition [21,22,23] and achieved encouraging results. 

### 2.2. Single Camera-Based Egocentric Activity Recognition

Egocentric activity recognition using a single wearable camera is commonly based on deep learning. As mentioned previously, the reported recognition methods can be divided into two main categories: video stream-based and photo stream-based [13]. The photo stream-based methods are usually applied to life loggers. As the camera in a life logger usually shoots at a very low frame rate (2–3 fpm)—the temporal correlation between the images in the photo stream is usually very low or there is no temporal correlation (only key frames are stored)—only high-level activities that last for a long time can be recognized. In this situation, we can only rely on CNN to learn the environmental information provided by individual images, and to complete the activity recognition by the similarity between the environmental information [9,13,24,25,26]. For example, Oliveira-Barra et al. [26] used a gradient boosting machine approach to retrieve activities based on their estimated relations with the recognized objects (by LSDA CNN) in the scene. When there is a certain temporal correlation between the images of a photo stream, an LSTM network can be added to the CNN for time correlation modeling. For example, Cartas et al. [13] proposed an end-to-end architecture consisting of LSTM units on the top of a VGG-16 CNN for each frame trained by processing the photo-streams using batches of a fixed size. Experimental results over the same dataset used in [26] have shown a 6% improvement in accuracy with respect to the VGG-16 baseline.

### 2.3. Hybrid Sensor System-Based Egocentric Activity Recognition 

With the continuous development of sensor technology, the performances of sensors have been improved, and their sizes and costs have decreased, resulting in hybrid sensor systems’ being easier to manufacture. In combinations of different types of sensors, as IMUs and cameras are more easily integrated within the same compact space, research on egocentric activity recognition based on IMU + camera hybrid systems have been actively conducted [2,14,27,28,29,30,31]. For example, Ozcan et al. [28] extracted histogram of gradient (HOG) and gradient local binary pattern (GLBP) features from the photo stream acquired by a wearable camera and fused them with the three-axis signal magnitude of an accelerometer to perform fall detection for the wearer. Experimental results show that, compared to accelerometer-only and camera-only methods, the fusion method not only has higher sensitivity but also reduces the number of false positives significantly. In [14], egocentric video and IMU data captured synchronously by Google Glass were used to recognize several egocentric activities. A multistream CNN and a multistream LSTM were used to learn the spatial and temporal features from videos and sensor streams, respectively, and the final recognition results were fused by maximum pooling. The fused data resulted in an average accuracy of 80.5% for 20 distinct activities, whereas the individual accuracies of video and sensor data were only 75% and 49.5%, respectively. These results show that, for egocentric activity recognition, it is beneficial to integrate motion sensors and cameras at both the hardware and algorithm levels.

Inspired by these related works, we propose a new hierarchical deep fusion framework for the IMU+camera hybrid sensor system, which enables the IMU and camera to work in their most suitable classification modes, thus achieving better recognition performance than single motion sensor-based and single camera-based methods.

## 3. The Proposed Approach

**Figure 1:**
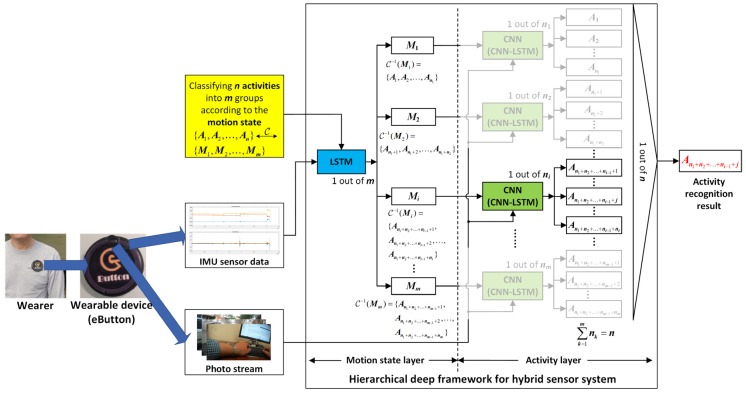
Overall architecture of the proposed hierarchical deep fusion framework.

The overall architecture of the proposed hierarchical deep activity recognition framework, which is applied to the IMU+camera wearable hybrid sensor system, is shown in Figure 1. The wearable device (eButton) is a hybrid sensor system, and the acquired vision data are a photo stream. The process of activity recognition is divided into two layers: a motion state layer and an activity layer. Before the egocentric data are sent to the proposed framework, all the activities to be recognized are first grouped according to their motion state through the correspondence, C. In all the groups, if a group contains only one activity to be recognized, it indicates that the activity can be directly recognized by IMU. In this case, the ADL is recognized directly in the motion state layer using the trained LSTM network. Conversely, if a group contains more than one activity to be recognized, which means that these activities are difficult to distinguish using only IMU data, they are further distinguished in the activity layer by photo stream through the pre-trained CNN (photo stream without time correlation) or CNN-LSTM (photo stream with time correlation).

### 3.1. Hierarchical Structure of the Activities to be Recognized

The results of existing work show that a motion sensor is only suitable for recognizing some dynamic activities with obvious differences in motion state, such as standing, sitting, walking, running, and climbing stairs. Motion sensors can achieve more than 90% accuracy in recognizing these activities [18]. In contrast, when the activities to be recognized include static activities without significant differences in motion state, such as reading, writing, and computer use, if the motion sensor data are used to distinguish all the activities to be recognized, the performance is usually poor. For example, when Sibo et al. [14] used motion sensor data to complete 20 kinds of activities (including ADLs with little difference in motion state and physical exercise activity with large difference in motion state, see Table 3) through multistream LSTM, the average recognition accuracy was only 49.5%. Therefore, the appropriate activity classification mode has a very important influence on the performance of motion sensor-based activity recognition.

In general, the activities of a human in daily life include both dynamic and static activities. Therefore, in the set of activities to be recognized, there must be some dynamic activities whose motion state is obviously different from that of other activities. Even if there is no separate dynamic activity, there must be more than one activity with the same motion state. For example, “writing” and “computer use” are both sedentary activities, and “cooking” and “sweeping” are both standing activities. We can group the activities to be recognized by using the similarity between the motion states of the activities. Even if a certain activity cannot be directly recognized, it can be classified into a certain activity group, thereby reducing the number of candidate activities for the subsequent photo stream-based classification process.

Let the set of activities to be recognized contain *n* activities, i.e., $A=\{A_{1},A_{2},\ldots ,A_{n}\}$, and let there be *m* motion states corresponding to all activities, i.e., $ℳ=\{M_{1},M_{2},\ldots ,M_{m}\}$. If each activity in A corresponds to only one motion state, then a mapping $\varphi_{A\to ℳ}$ from A to ℳ can be defined, such that

(1) $$\varphi_{A\to ℳ}(A):\;A\to ℳ,\;A\in A.$$

**Figure 2:**
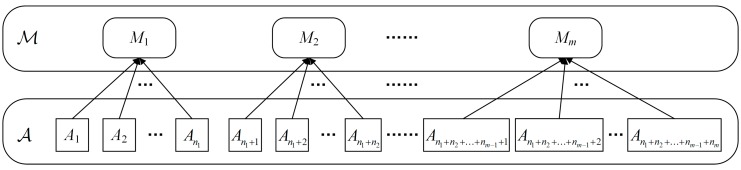
Hierarchical relationship of the mapping $\varphi_{A\to ℳ}$.

As the mapping results of multiple activities may be the same, i.e., multiple activities have the same motion state (*n* > *m*), A can also be rewritten into a group form using $\varphi_{A\to ℳ}^{-1}$, as in Equation (2):

(2) $$A=\{\underset{\varphi_{A\to ℳ}^{-1}(M_{1})}{\underset{︸}{A_{1},A_{2},\ldots ,A_{n_{1}}}},\underset{\varphi_{A\to ℳ}^{-1}(M_{2})}{\underset{︸}{A_{n_{1}+1},A_{n_{1}+2},\ldots ,A_{n_{1}+n_{2}}}},\underset{\ldots }{\underset{}{\ldots }},\underset{\varphi_{A\to ℳ}^{-1}(M_{m})}{\underset{︸}{A_{n_{1}+n_{2}+\ldots +n_{m-1}+1,}A_{n_{1}+n_{2}+\ldots +n_{m-1}+2},\ldots ,A_{n_{1}+n_{2}+\ldots +n_{m-1}+n_{m}}}}\}$$

where *n_i_* (*i =* 1, 2, 3, …, *m*) is the number of activities in each group and should satisfy $\sum_{i=1}^{m}n_{i}=n$. As each activity corresponds to only one motion state, there is no repetitive activity in each group of ℳ; i.e., for ∀i,j=1,2,…,m and i≠j, there should be $\varphi^{-1}(M_{i})\cap \varphi^{-1}(M_{j})=\emptyset$ and $\overset{m}{\underset{k=1}{\cup }}\varphi^{-1}(M_{k})=A$. The hierarchical relationship of the mapping $\varphi_{A\to ℳ}$ is shown in Figure 2. 

It should be pointed out that, in actual situations, some activities may have multiple motion states. For example, “reading” can be done while sitting or standing, and “making phone calls” can be done either by sitting, standing, or by walking. In order to make the above hierarchical structure applicable to activities that may have multiple motion states, we can add the activity to all groups of possible motion states corresponding to this activity. Thus, a many-to-one mapping $\varphi_{A\to ℳ}$ between A and ℳ is extended to a many-to-many correspondence $C_{A↔ℳ}$. At this point, ∃i,j=1,2,…,m and i≠j, there should be $C^{-1}(M_{i})\cap C^{-1}(M_{j})\neq \emptyset$, but $\overset{m}{\underset{k=1}{\cup }}C^{-1}(M_{k})=A$ still holds. Meanwhile, the number of activities in each group should satisfy $\sum_{i=1}^{m}n_{i}>n$.

With the hierarchical structure established by $\varphi_{A\to ℳ}$ or $C_{A↔ℳ}$, the hierarchical recognition of the activity to be recognized can be completed, i.e., the activity to be recognized first enters the group according to its motion state, and the specific activity is then recognized in the group with the narrowed candidate range. In this paper, for the egocentric data acquired by the self-developed chest-worn life logger, i.e., eButton, the activity set A is defined as

(3) $$\begin{array}{ll} A= & \{A_{1},A_{2},\ldots ,A_{15}\}=\\ & \{ʺ\mathrm{computer use},ʺ\;ʺ\mathrm{eating},ʺ\;ʺ\mathrm{entertainment},ʺ\;ʺ\mathrm{meeting},ʺ\;ʺ\mathrm{nap},ʺ\;ʺ\mathrm{reading},ʺ\;ʺ\mathrm{shopping},ʺ\;ʺ\mathrm{sweeping},ʺ\\ & ʺ\mathrm{talking},ʺ\;ʺ\mathrm{telephone}\;\mathrm{use},ʺ\;ʺ\mathrm{transportation},ʺ\;ʺ\mathrm{walking}\;\mathrm{outside},ʺ\;ʺ\mathrm{washing}\;\mathrm{up},ʺ\;ʺ\mathrm{watching}\;\mathrm{TV},ʺ\;ʺ\mathrm{writing}ʺ\}=\\ & \{ʺ\mathrm{CU},ʺ\;ʺ\mathrm{ET},ʺ\;ʺ\mathrm{EM},ʺ\;ʺ\mathrm{MT},ʺ\;ʺ\mathrm{NP},ʺ\;ʺ\mathrm{RD},ʺ\;ʺ\mathrm{SP},ʺ\;ʺ\mathrm{SW},ʺ\;ʺ\mathrm{TK},ʺ\;ʺ\mathrm{TU},ʺ\;ʺ\mathrm{TP},ʺ\;ʺ\mathrm{WO},ʺ\;ʺ\mathrm{WU},ʺ\;ʺ\mathrm{TV},ʺ\;ʺ\mathrm{WT}ʺ\} \end{array}$$

Considering the motion states corresponding to all the activities in A, the motion state set ℳ is determined as

(4) $$ℳ=\{M_{1},M_{2},M_{3},M_{4}\}=\{ʺ\mathrm{lying},ʺ\;ʺ\mathrm{sedentary},ʺ\;ʺ\mathrm{standing},ʺ\;ʺ\mathrm{walking}ʺ\}=\{ʺ\mathrm{LY},ʺ\;ʺ\mathrm{SD},ʺ\;ʺ\mathrm{ST},ʺʺ\mathrm{WK}ʺ\}.$$

As some of the activities in A have multiple motion states, such as “reading,” “talking,” and “telephone use,” it is necessary to define the correspondence between A and ℳ by $C_{A↔ℳ}$, i.e.,

(5) $$\left\{ \begin{array}{l} {C^{-1}}_{A↔ℳ}(ʺ\mathrm{LY}ʺ)=\{ʺ\mathrm{NP},ʺ\;ʺ\mathrm{TU}ʺ\}\\ {C^{-1}}_{A↔ℳ}(ʺ\mathrm{SD}ʺ)=\{ʺ\mathrm{CU},ʺ\;ʺ\mathrm{ET},ʺ\;ʺ\mathrm{EM},ʺ\;ʺ\mathrm{MT},ʺ\;ʺ\mathrm{RD},ʺ\;ʺ\mathrm{SP},ʺ\;ʺ\mathrm{TK},ʺ\;ʺ\mathrm{TU},ʺ\;ʺ\mathrm{TP},ʺ\;ʺ\mathrm{TV},ʺ\;ʺ\mathrm{WT}ʺ\}\\ {C^{-1}}_{A↔ℳ}(ʺ\mathrm{ST}ʺ)=\{ʺ\mathrm{ET},ʺ\;ʺ\mathrm{EM},ʺ\;ʺ\mathrm{RD},ʺ\;ʺ\mathrm{SP},ʺ\;ʺ\mathrm{SW},ʺ\;ʺ\mathrm{TK},ʺ\;ʺ\mathrm{TU},ʺ\;ʺ\mathrm{TP},ʺ\;ʺ\mathrm{WU}ʺ\}\\ {C^{-1}}_{A↔ℳ}(ʺ\mathrm{WK}ʺ)=\{ʺ\mathrm{SP},ʺ\;ʺ\mathrm{WO}ʺ\} \end{array}\right..$$

**Figure 3:**
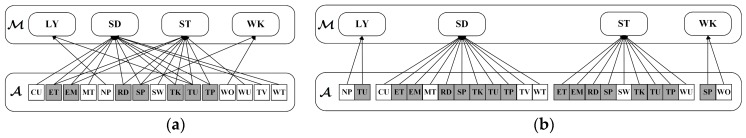
Hierarchical relationship of the correspondence $C_{A↔ℳ}$ defined by Equation (5). (**a**) is the original correspondence of $C_{A↔ℳ}$; (**b**) is the converted group correspondence of $C_{A↔ℳ}$.

According to Equation (5), the hierarchy and correspondence of $C_{A↔ℳ}$ is shown in Figure 3a, and the activity in the dark square has multiple motion states. Meanwhile, we can convert the corresponding relation represented in Figure 3a into a group correspondence based on motion state by the activity repetition. The converted group correspondence is shown in Figure 3b.

### 3.2. LSTM in the Motion Sensor Layer

LSTM, originally proposed by Hochreiter et al. [32], is a special type of recurrent neural network (RNN) that can be used to learn data with long-term correlation. Compared with traditional RNN, LSTM provides a solution by incorporating memory units that allow the network to learn when to forget previous hidden states and when to update hidden states given new information. LSTM has achieved considerable success in many aspects of timing signal processing, including motion sensor-based activity recognition. In the hierarchical framework proposed in this paper, LSTM is used to classify the motion state of the motion sensor data in the motion sensor layer, as shown in Figure 1. Specifically, we use the minor simplified LSTM proposed by Graves et al. [33]. This LSTM network has been used by many researchers for human activity classification and recognition [34,35,36].

**Figure 4:**
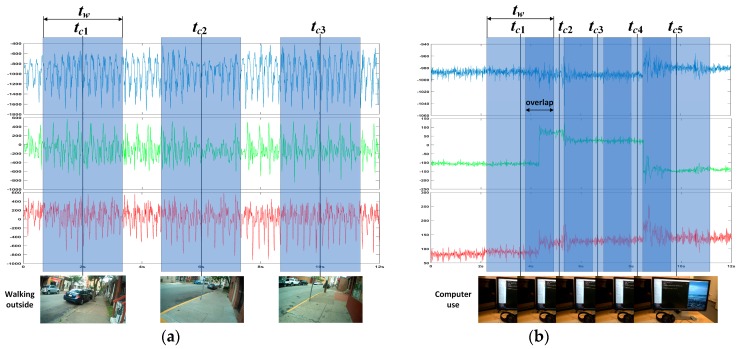
Schematic diagram of the correspondence between sensor data and images (or image sequences): (**a**) When the sampling rate of images is low, there is no overlap between the time windows. (**b**) When the sampling rate of the images is higher and the time window is wider, overlap occurs between the time windows.

After the sensor data are classified by LSTM, the pre-trained CNN network is required to further classify the images (or image sequence) corresponding to the sensor data in each group. Therefore, there should be a correspondence between the sensor data and the images (or image sequence). In order to achieve the correspondence between the sensor data and the images/image sequence, we take the shooting time *t_c_* of each frame in the photo stream as the center and take a time window with a fixed width of *t_w_*, as shown in Figure 4a. All the sensor data in the window are the sensor data corresponding to the image and can be used as the input of the LSTM network to complete the training and motion state classification. It should be noted that, when the sampling rate of the photo stream is higher and *t_w_* is wider, there may be a time window overlap, as shown in Figure 4b.

In addition, when the LSTM network is used to classify the input temporal sequence, a fully connected layer (FC layer) and a softmax layer need to be added to the LSTM layer. Therefore, if the input sequence is set as $x=(x_{1},x_{2},\ldots ,x_{T})$ and the output classification result is *y*, *y* can be represented as

(6) $$y=softmax_{FC}(LSTM(x))=softmax_{FC}(LSTM(\{x_{1},x_{2},\ldots ,x_{T}\})).$$

### 3.3. CNN or CNN-LSTM in the Activity Layer

In the proposed deep recognition framework, after grouping the activity according to motion state using IMU sensor data, if the group contains multiple activities to be recognized, such as the grouping result of the eButton Dataset shown in Equation (5), the activities in each group will be further recognized by the photo stream through the deep CNN network. When the images in the photo stream are recorded at a low frame rate, the temporal correlation between the images is very low or there is no temporal correlation, so the features will be directly extracted and classified through the pre-trained deep CNN to complete the activity recognition. While the images in the photo stream are recorded at a high frame rate, there is a temporal correlation between the images. In this case, the spatial features of each frame in the photo stream will be extracted by using a pre-trained deep CNN, and the spatial features will then be sent to an LSTM network so that the activity recognition based on spatio-temporal features is accomplished by using the CNN-LSTM deep network framework.

**Figure 5:**
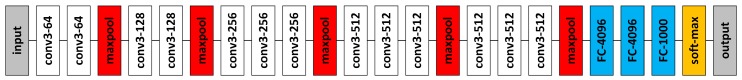
Architecture of the VGG-16 network.

It is well known that the classification performance of deep neural networks depends not only on the network structure but also on the number of training samples. For egocentric activity recognition, existing public datasets, such as UCF11 [37], have not only a small number of samples but also a limited number of activities, so they are difficult to directly use to obtain well-trained CNN models for activity recognition with good performance. In order to overcome the problem of limited training, motivated by Simonyan et al. [38], we use the VGG-16 model [39], which was well-trained on ImageNet [40] and has achieved great success in the field of image classification, to extract the spatial features of each frame in the photo stream. The architecture of the VGG-16 network is shown in Figure 5.

It should be pointed out that, when using pre-trained networks such as VGG-16 and AlexNet, there are problems such as excessive parameter size and easy over-fitting because the concatenated FC layers are between the convolutional layer and the softmax layer. To solve these problems, Min et al. [41] proposed a Network In Network (NIN) architecture, which replaces the traditional fully connected layer with global average pooling (GAP) after the convolutional layer, and takes the average of each feature map so that the resulting vector is fed directly into the softmax layer. Recently, the NIN architecture has achieved success in many applications in the fields of image classification and recognition, video expression, and expression recognition [42,43,44]. Referring to these successful applications, based on the VGG-16 network, we use GAP instead of concatenated FC layers to obtain feature maps for each activity, and then use these features to finetune a single FC layer for subsequent activity classification. 

**Figure 6:**
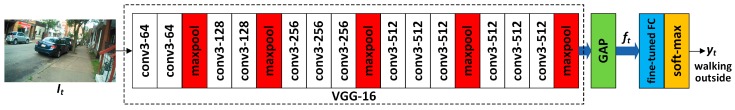
Architecture of the proposed fine-tuned CNN for low-frame-rate photo streams.

When the fine-tuned network is used for the low-frame-rate photo stream, the structure of the network is as shown in Figure 6. In Figure 6, if a certain frame in a low-frame-rate photo stream is set as $I_{t}$, the deep convolution operation is first performed by using the cascaded convolution layers of the VGG-16 network, and the output of the last convolutional layer is set as $VGG_{conv}(I_{t})$. Next, GAP is used to obtain the feature maps for classification, denoted as $f_{t}^{S}=GAP(VGG_{conv}(I_{t}))$. These feature maps are then fed into the fine-tuned FC layer, and the final activity classification is completed by the softmax layer. The classification result $y_{t}$ can be expressed as follows:

(7) $$y_{t}=softmax_{FC}(f_{t}^{S})=softmax_{FC}(GAP(VGG_{conv}(I_{t}))).$$

**Figure 7:**
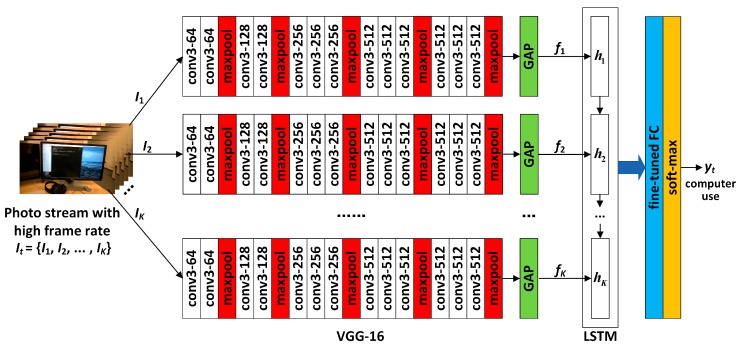
Architecture of the proposed CNN-LSTM network for high-frame-rate photo streams.

When the network is used for high-frame-rate photo streams, the structure is as shown in Figure 7. Let a certain image sequence $I_{t}$ corresponding to a certain activity in the photo stream contain *K* frames, i.e., $I_{t}=\{I_{1},I_{2},\ldots ,I_{K}\}$. First, the deep convolution operation is performed on each frame through the VGG-16 network, and the convolution result of each frame is then converted into a spatial feature map by using GAP, which is denoted as $f_{t}^{S}=GAP(VGG_{conv}(I_{t}))=\{f_{1},f_{2},\ldots ,f_{K}\}$. Next, $f_{t}^{S}$ is fed into a single-layer LSTM network to obtain the spatio-temporal features of sequence $I_{t}$, denoted as $f_{t}^{S-T}=LSTM(f_{t}^{S})$. The spatio-temporal features are then fed into the fine-tuned FC layer so that activity classification is finally completed by the softmax layer, i.e.,

(8) $$y_{t}=softmax_{FC}(f_{t}^{S-T})=softmax_{FC}(LSTM(f_{t}^{S}))=softmax_{FC}(LSTM(\{f_{1},f_{2},\ldots ,f_{K}\})).$$

### 3.4. Combination of Motion Sensor Data and Photo Stream

From the above, as the photo stream-based recognition is mainly based on the pre-trained VGG-16 CNN, in practical application of the method proposed in this paper, the networks that require prior training are as follows (assuming that the activities to be recognized can be divided into *m* groups according to the motion state): (1) The LSTM network for IMU sensor data classification, defined as $LSTM^{x}$. (2) For low-frame-rate photo streams, the fine-tuned FC layer for single frame classification in each group, defined as $FC_{j}^{I},\;j=1,2,\ldots ,m$. (3) For high-frame-rate photo streams, the LSTM and the corresponding fine-tuned FC layer for extracting spatio-temporal features from the image sequence in each of the *m* groups are defined as $LSTM_{j}^{I},\;j=1,2,\ldots ,m$ and $FC_{j}^{LSTM},\;j=1,2,\ldots ,m$, respectively. 

**Figure 8:**
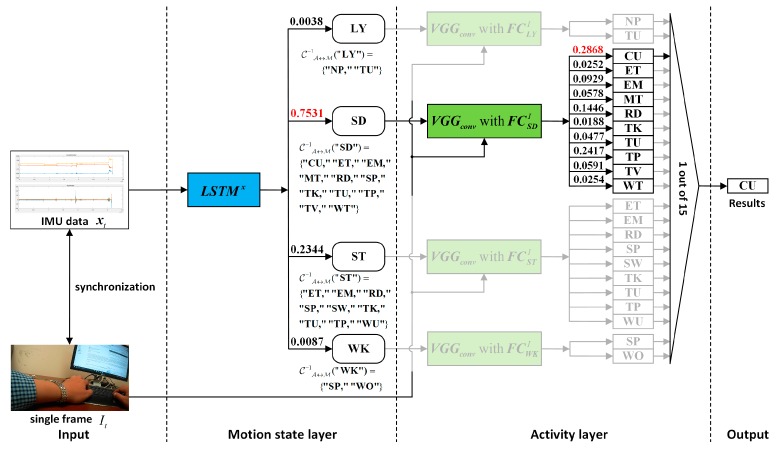
A specific fusion example of the proposed hierarchical deep fusion framework applied to the self-built eButton hybrid dataset.

After the training of all networks that require prior training is completed, the recognition results of IMU sensor data and photo stream can be fused based on the hierarchical deep fusion framework shown in Figure 1. The specific algorithm flow is presented in Algorithm 1. A specific fusion example illustrating the application of the proposed hierarchical deep fusion framework to the self-built eButton hybrid dataset is presented in Figure 8. 

**Algorithm 1**: Algorithm flow of the proposed hierarchical deep fusion framework.Input: activity sets $A=\{A_{1},A_{2},\ldots ,A_{n}\}$; motion state sets $ℳ=\{M_{1},M_{2},\ldots ,M_{m}\}$; correspondence $C_{A↔ℳ}$;  IMU sequence $x_{t}$; single frame $I_{t}$ or image sequence $I_{t}$ (corresponding to $x_{t}$); pre-trained $LSTM^{x}$ (for $x_{t}$); pre-trained $VGG_{conv}$; pre-trained $FC_{j}^{I},j=1,2,\ldots ,m$ (for $I_{t}$); pre-trained $LSTM_{j}^{I},j=1,2,\ldots ,m$ (for $I_{t}$); pre-trained $FC_{j}^{LSTM},j=1,2,\ldots ,m$ (for $LSTM_{j}^{I}$); indicator of the photo stream *Ips* (*H* or *L*) Output: activity index k,k∈{1,2,…,n}
(1)Input $x_{t}$ into $LSTM^{x}$(2)Get the grouping index *j* of $x_{t}$ with Equation (6)(3)If *Ips==L* // *Input photo stream is a low-frame-rate photo stream (*$x_{t}$ *corresponds to single frame* $I_{t}$) (4) Input $I_{t}$ into $VGG_{conv}$ with $FC_{j}^{I}$        //*CNN as shown in Figure 6*(5) Get *k* with Equation (7) (6)Else   // *Input photo stream is a high-frame-rate photo stream (*$x_{t}$ *corresponds to image sequence* $I_{t}$) (7) Input $I_{t}$ into $VGG_{conv}+LSTM_{j}^{I}$ with $FC_{j}^{LSTM}$  //*CNN+LSTM as shown in Figure 7*
(8) Get *k* with Equation (8) (9)end If


## 4. Experimental Evaluation

We used two datasets obtained by the hybrid sensor system to evaluate the performance of the proposed hierarchical deep fusion framework. The two datasets are the eButton egocentric activity dataset (hereinafter referred to as the eButton Dataset) obtained by the self-developed life logger eButton, and the multimodal egocentric activity dataset (hereinafter referred to as the Multimodal Dataset) that Sibo et al. established in [14] with Google Glass. In the evaluation, in addition to the proposed hierarchical deep fusion framework, the existing direct fusion algorithm was also applied to the above two datasets to complete the performance comparison.

### 4.1. Datasets

Although there are many public datasets for egocentric activity recognition, most of them were constructed using single camera/sensor data. Very few public datasets comprise data collected using hybrid sensor systems, especially “camera+sensors” systems. In this study, we used a public dataset and a self-built dataset to complete the algorithm evaluation. Both datasets are “camera+sensors” hybrid datasets.

#### 4.1.1. The eButton Dataset

**Figure 9:**
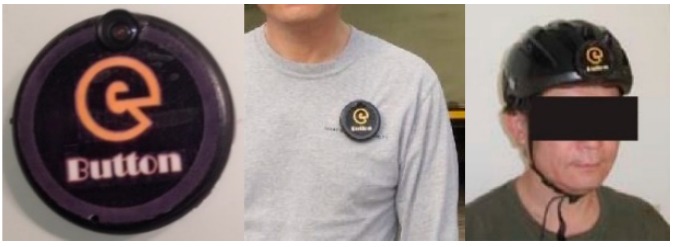
Appearance and two possible ways to wear the eButton device.

Previously, our laboratory developed eButton (as shown in Figure 9), a disc-like wearable life logger the size of an Oreo cookie, for studying human diet, physical activity, and sedentary behavior [15,45]. eButton is equipped with a set of sensors, including a camera, an IMU, and other sensors that were not used in the current study, for measuring the temperature, lighting, and atmospheric pressure. The camera has a resolution of 1280 × 720 pixels. To save power, the camera acquires one image every four or more seconds. The built-in IMU contains a three-axis accelerometer and a three-axis gyroscope, both of which have a sampling frequency of 90 Hz.

Two volunteers with regular daily routines and relatively invariant living environments were selected for our experiments. The volunteers wore eButton for relatively long periods (~10 h/day for around three months). To form a gold standard for performance comparison, the resulting egocentric data were manually reviewed and annotated. Specifically, (1) for the photo stream, activity set A is first determined according to the application requirement and the activity pattern of the wearer. Each frame in the photo stream is then manually inserted into its corresponding activity group in A. Thus, each frame in the photo stream has a unique group number as its label. Considering that the frequency and duration of the different activities vary widely, there will be a large imbalance among the number of frames in different activity groups. A key frame extraction method proposed in [46,47] is used in groups with an excessive number of frames to make the groups relatively balanced. (2) For the IMU data, the IMU time segment corresponding to each frame in the photo stream can be obtained by using the corresponding method shown in Figure 4, where *t_c_* is the time stamp of each frame, and *t_w_* is given in advance. Thus, these IMU time segments have the same activity group labels as their corresponding frames. (3) When constructing the training set and test set for motion state set ℳ, after changing A in Step (1) to ℳ, and then repeating Steps (1) and (2), the motion state group label of each frame and its corresponding IMU segment can be obtained.

**Figure 10:**
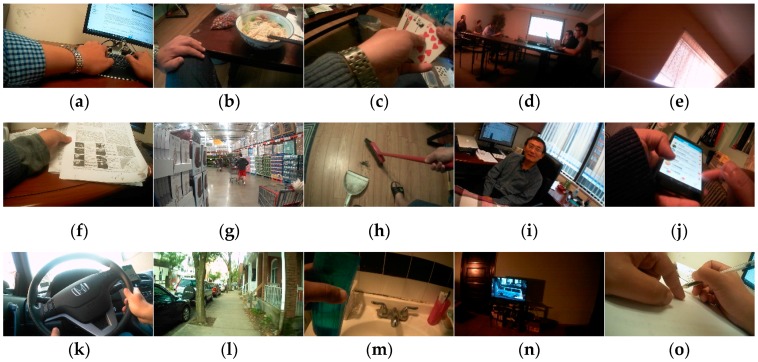
Example image of each activity in the training set. Images (**a**–**o**) correspond to CU, ET, EM, MT, NP, RD, SP, SW, TK, TU, TP (driving), WO, WU, TV, and WT, respectively.

As the two eButton wearers participated in the study for about three months, we had sufficient data to form two independent datasets, one for training and the other for testing. Table 1 lists the number of time segments used for the grouping of IMU sensor data. Among them, each time segment is a six-dimensional vector composed of three-axis accelerometer data and three-axis gyroscope data, and all the time segments are grouped manually according to the motion state set ℳ defined by Equation (4). Table 2 lists the number of images in the training and test sets. Among them, all the images are classified manually according to the activity set A shown in Equation (3). Figure 10 shows example images for the 15 ADLs in the training set. It should be noted that the correspondence between the two test sets in Table 1 and Table 2 has been established according to $C_{A↔ℳ}$ shown in Equation (5) to form a hybrid dataset. In the corresponding process, *t_c_* of the time segment of each sensor is the time stamp of the image and *t_w_* = 3 s. As the maximum shooting frame rate of the eButton camera is 1/4 s, there is no overlap in the time window.

#### 4.1.2. The Multimodal Dataset

The Multimodal Dataset is publicly available at http://people.sutd.edu.sg/˜1000892/dataset. The dataset contains 20 distinct life-logging activities performed by different human subjects. The data were captured using Google Glass, which records high-quality synchronized video and sensor streams. The dataset has 200 sequences in total, and each activity category has 10 sequences of 15 seconds each. The categories of egocentric activity are presented in Table 3. Furthermore, the categories can also be grouped into four top-level types: Ambulation, Daily Activities, Office Work, and Exercise. As the dataset contains both egocentric video and sensor data recorded simultaneously, it can be used to evaluate the hybrid approaches.

When the proposed framework is applied to the Multimodal Dataset, as the dataset only contains video stream, whereas the proposed framework is based on photo stream, it was necessary to convert the video to photo stream and associate the photo stream with the sensor data to construct the hybrid dataset. In the construction of the hybrid dataset, the composition of the sensor data is consistent with that in [14], i.e., accelerometer (three-axis), gyroscope (three-axis), magnetic field (three-axis), and rotation vectors (three-axis and magnitude). In order to make the data structure of the motion sensor consistent with the eButton Dataset, the window width $t_{w}$ of the time segment was also selected as 3 s, and the interval of the time segment center $t_{c}$ was chosen to be 1 s. Thus, the sensor data corresponding to each 15 s video could be divided into 15 overlapping time segments. Accordingly, each video was also converted into 15 overlapping 3 s photo streams. As the video in the dataset was recorded at a high frame rate (about 30 fps), there is a strong temporal correlation between the images in each 3 s photo stream (about 90 images). The converted hybrid dataset contains a total of 200 × 15 = 3000 3 s time segments for sensor data and corresponding 3000 3 s photo streams. For each activity to be recognized, the hybrid data are 150 3 s time segments and corresponding 150 3 s photo streams.

In addition, as the activity set A shown in Table 3 changes significantly with respect to the eButton Dataset, as shown in Equation (3), and the motion states corresponding to the activities are also different, it is necessary to redefine the motion state set ℳ, as well as $\varphi_{A\to ℳ}$ or $C_{A↔ℳ}$. Note that the data in the Multimodal Dataset are recorded by many different wearers wearing Google Glass, and different wearers have different activity habits, so the same motion state of different wearers is difficult to define. Thus, we use fine and coarse methods to first give the initial motion state set ${ℳ}_{init}$ and the initial correspondence $C_{A↔ℳ}^{init}$ according to the motion states of all the activities in the activity set A, i.e.,

(9) $$\begin{array}{ll} {ℳ}_{init}=\{M_{1},M_{2},\ldots ,M_{10}\}= & \{ʺ\mathrm{walking},ʺ\;ʺ\mathrm{walking}\;\mathrm{upstairs},ʺ\;ʺ\mathrm{walking}\;\mathrm{downstairs},ʺ\;ʺ\mathrm{riding},ʺ\;ʺ\mathrm{sedentary},ʺ\\ & ʺ\mathrm{standing},ʺ\;ʺ\mathrm{running},ʺ\;ʺ\mathrm{doing}\;\mathrm{push-ups},ʺ\;ʺ\mathrm{doing}\;\mathrm{sit-ups},ʺ\;ʺ\mathrm{cycling}ʺ\}\\ & =\{ʺ\mathrm{WK},ʺ\;ʺ\mathrm{WK-US},ʺ\;ʺ\mathrm{WK-DS},ʺ\;ʺ\mathrm{RD},ʺ\;ʺ\mathrm{SD},ʺ\;ʺ\mathrm{ST},ʺ\;ʺ\mathrm{RN},ʺ\;ʺ\mathrm{DPU},ʺ\;ʺ\mathrm{DSU},ʺ\;ʺ\mathrm{CY}ʺ\} \end{array}$$

(10) $$\left\{ \begin{array}{l} \ldots \\ {C^{(init)-1}}_{A↔ℳ}(ʺ\mathrm{RD}ʺ)=\{ʺ\mathrm{RD-VU},ʺ\;ʺ\mathrm{RD-VD},ʺ\;ʺ\mathrm{RD-SU},ʺ\;ʺ\mathrm{RD-SD}ʺ\}\\ {C^{(init)-1}}_{A↔ℳ}(ʺ\mathrm{SD}ʺ)=\{ʺ\mathrm{SI},ʺ\;ʺ\mathrm{ET},ʺ\;ʺ\mathrm{DR},ʺ\;ʺ\mathrm{TX},ʺ\;ʺ\mathrm{MP},ʺ\;ʺ\mathrm{PC},ʺ\;ʺ\mathrm{RD},ʺ\;ʺ\mathrm{WT},ʺ\;ʺ\mathrm{OF}ʺ\}.\\ {C^{(init)-1}}_{A↔ℳ}(ʺ\mathrm{ST}ʺ)=\{ʺ\mathrm{ET},ʺ\;ʺ\mathrm{DR},ʺ\;ʺ\mathrm{TX},ʺ\;ʺ\mathrm{MP},ʺ\;ʺ\mathrm{RD},ʺ\;ʺ\mathrm{OF}ʺ\}\\ \ldots \end{array}\right.$$

Equation (10) omits the grouping correspondence in which the motion state is consistent with the corresponding activity (i.e., there is only one activity in the grouping). On the basis of ${ℳ}_{init}$ and $C_{A↔ℳ}^{init}$, the grouping method is adjusted according to the measured results, and the indistinguishable motion states are merged. During the adjustment of the grouping method, multiple grouping methods shown in Table 4 were established. In Table 4, ${ℳ}_{6}$ is the final adopted grouping method for detailed performance evaluation and comparison with other algorithms, and the remaining grouping methods are used to evaluate the impact of different grouping methods on the performance of the proposed framework. 

### 4.2. Experimental Setup

We implemented the proposed hierarchical deep fusion framework on the Keras+Tensor Flow (tensorflow-gpu==1.6) platform running on Ubantu and a Nvidia TitanX (Pascal) GPU. The definitions of A, ℳ, and $C_{A↔ℳ}$ used during application of the proposed framework to the eButton Dataset are shown in Equations (3)–(5). For the proposed framework’s use with the Multimodal Dataset, A is shown in Table 3, and ℳ and $C_{A↔ℳ}$ are defined as shown in Table 4, where ${ℳ}_{6}$ is used for detailed performance evaluation and comparison with other algorithms, and the remaining grouping methods are used to evaluate the impact of different grouping methods on the performance of the proposed framework. In the eButton Dataset, the training set and the test set are given, respectively (as shown in Table 1 and Table 2), which are the egocentric hybrid data of the same wearer at different times. In contrast, there is no clear distinction between the training set and the test set in the Multimodal Dataset. We adopted the same method in [14] to divide the 200 sequences into 10 splits (each split containing 20 sequences, each of which corresponds to an activity to be recognized). Thus, the training set and test set were determined by the leave-one-out cross-validation method, and the average accuracy of 10 tests was taken as the final accuracy. In addition, the *F*_1_ measure [48] was selected as the criterion for evaluating the different classification methods, which is commonly used in the field of pattern recognition. The *F*_1_ measure is defined as

(11) $$\begin{array}{l} F_{1}=2\cdot PR/(P+R)\\ P=TP/(TP+FP),\;R=TP/(TP+FN) \end{array}$$

where *P* is precision, and *R* is recall. *TP*, *FP*, and *FN* represent the number of true positive samples, false positive samples, and false negative samples, respectively, derived from the confusion matrix. *F*_1_ is also called the harmonic mean of recall and precision.

The $LSTM^{x}$ to classify the motion state of the two datasets have an almost identical structure and parameters: an LSTM layer containing 128 hidden units, followed by an FC layer determined by the number of elements of ℳ and a softmax layer to complete the classification prediction. In the network training, the optimization method selected was adaptive moment estimation (Adam) for 150 epochs for all folds and the batch size was set to 30. The learning rate was set as 0.001. The exponential decay rate of the first moment estimation was 0.9, and the second moment estimation was 0.99.

As the photo streams in the eButton Dataset are captured at a low frame rate, in the process of classifying the activities in the grouping by using the photo stream, the fine-tuned VGG-16 architecture shown in Figure 6 was adopted. We froze all layers of base_model so that the bottleneck feature could be obtained correctly. The optimization method was Adam for 50 epochs for all folds. The batch size was set to eight, the learning rate to 0.001, the exponential decay rate of the first moment estimation to 0.9, and the second moment estimation to 0.99.

The 3 s photo streams in the Multimodal Dataset are high-frame-rate sequential photo streams. In the process of classifying the activities in the grouping, the fine-tuned VGG16-LSTM architecture shown in Figure 7 was adopted. Among them, the structure and parameters of VGG-16 were the same as those applied to the eButton Dataset. All LSTM networks after VGG-16, i.e., $LSTM_{j}^{I},j=1,2,\ldots ,m$, contained 512 hidden units and a 512-unit fully connected layer, and their dropout parameters were all set to 0.7. The other parameters of $LSTM_{j}^{I}$ were the same as $LSTM^{x}$.

### 4.3. Experimental Results

**Figure 11:**
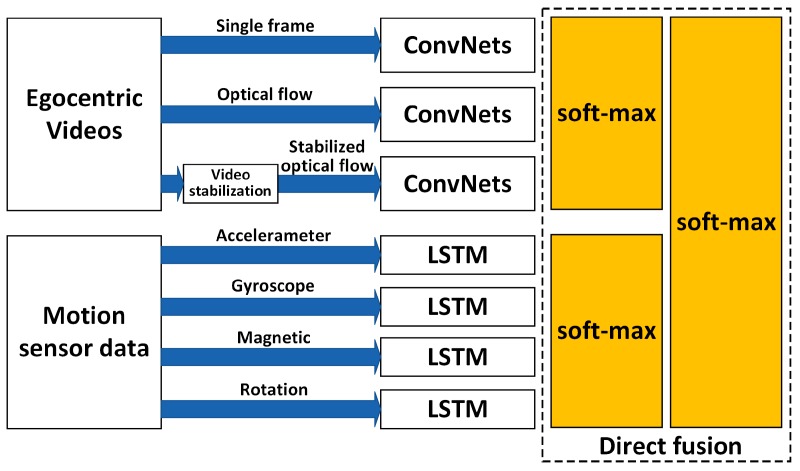
Architecture of the multistream direct fusion method proposed in [14].

The proposed hierarchical deep fusion framework separately processes the motion sensor data and the photo stream. This results in the classification result of the motion sensor and the classification result of the photo stream both having an influence on the final recognition result. Therefore, the algorithm was evaluated using single-sensor data-based recognition results, single-photo stream-based recognition results, and fusion results. Further, in order to verify the performance of the hierarchical fusion, the multistream direct fusion method proposed by Sibo et al. [14] was compared with the proposed deep fusion framework. The architecture of the multistream direct fusion method proposed by Sibo et al. [14] is shown in Figure 11. In the part where fusion of motion sensor-based recognition results and video-based recognition results occurs, the output of the networks (ConvNets and LSTM) are directly fused in the softmax layer with average pooling or maximum pooling.

#### 4.3.1. Results on the eButton Dataset

**Figure 12:**
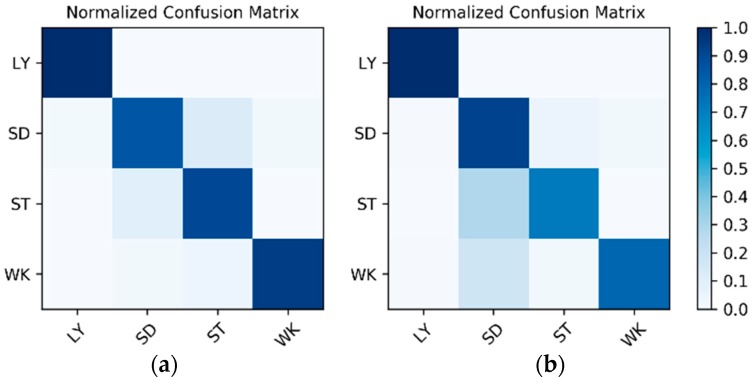
Confusion matrices for the classification results of the inertial measurement unit (IMU) sensor data: (**a**) W1; (**b**) W2.

**Results on the IMU sensor data:** The dataset for the IMU sensor data classification test is shown in Table 1. As the dataset contains the data of two wearers, the experimental results also distinguish between the two wearers (W1 and W2). The confusion matrices for the classification results of the test set in Table 1 are shown in Figure 12. The *F*_1_ accuracy is shown in Table 5.

**Figure 13:**
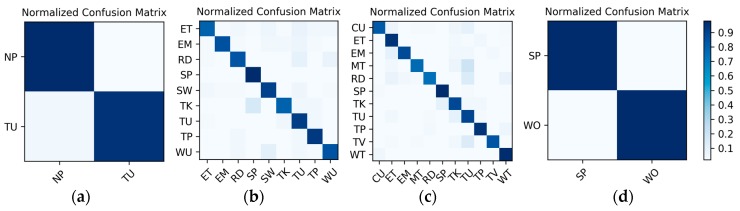
W1’s confusion matrices for the output of the $VGG_{conv}$ corresponding to each group: (**a**) lying; (**b**) sedentary; (**c**) standing; (**d**) walking.

**Figure 14:**
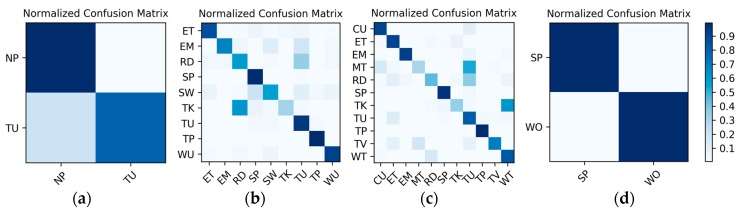
W2’s confusion matrices for the output of the $VGG_{conv}$ corresponding to each group: (**a**) lying; (**b**) sedentary; (**c**) standing; (**d**) walking.

**Results on the low frame rate photo stream:** The dataset for the low frame rate photo stream classification test is shown in Table 2. As the photo stream is classified in the grouping defined by Equations (4) and (5) in the proposed hierarchical framework, the dataset should also be adjusted according to the grouping. The fine-tuned VGG-16 network shown in Figure 6 was set up in each of the four groups to complete the training and testing. In addition, similar to the IMU sensor data, the experimental results also distinguish the two wearers W1 and W2. The confusion matrices for the output of the $VGG_{conv}$ corresponding to each group are shown in Figure 13 and Figure 14. The *F*_1_ accuracies for each group are shown in Table 6, Table 7, Table 8 and Table 9.

**Figure 15:**
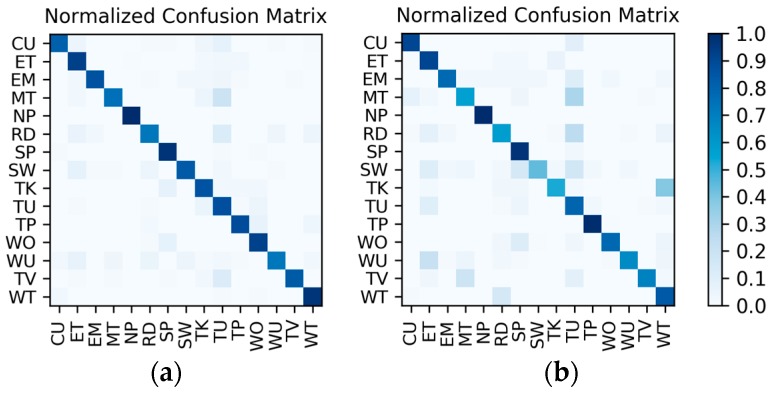
Confusion matrices for the hierarchical fusion results: (**a**) W1; (**b**) W2.

**Results of the hierarchical fusion:** After the training of $LSTM^{x}$ and fine-tuned $VGG_{conv}$ is completed, the sensor-based recognition results and the photo stream-based recognition results can be fused by the hierarchical framework shown in Figure 8. The confusion matrices after fusion for W1 and W2 are shown in Figure 15. The *F*_1_ accuracy is shown in Table 10.

**Figure 16:**
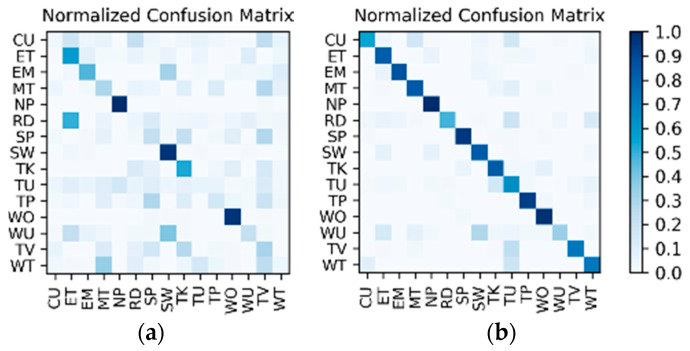
W1’s confusion matrix using a single sensor: (**a**) IMU; (**b**) photo stream.

**Figure 17:**
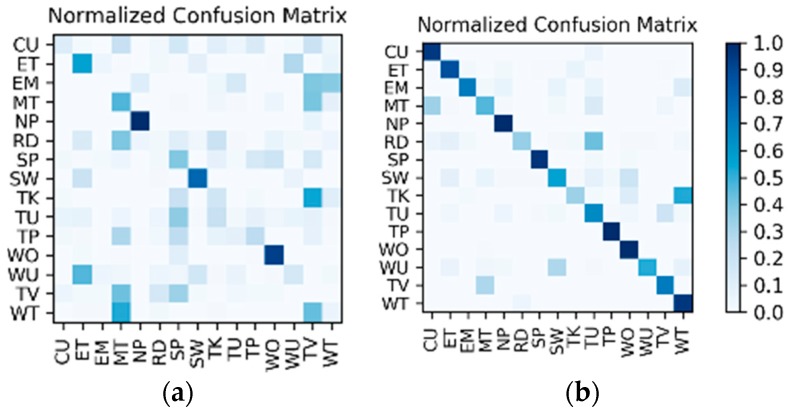
W2’s confusion matrix using a single sensor: (**a**) IMU; (**b**) photo stream.

**Figure 18:**
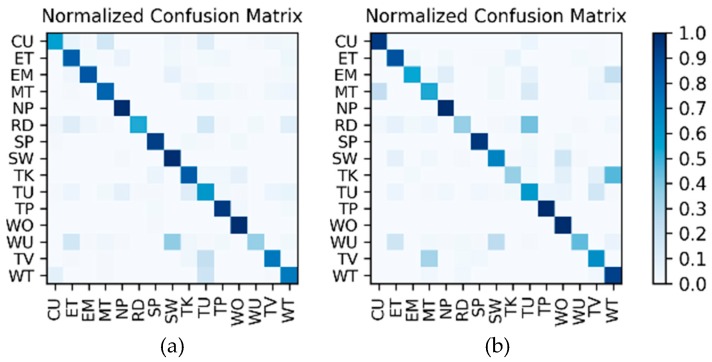
Confusion matrices for the direct fusion results: (**a**) W1; (**b**) W2.

**Figure 19:**
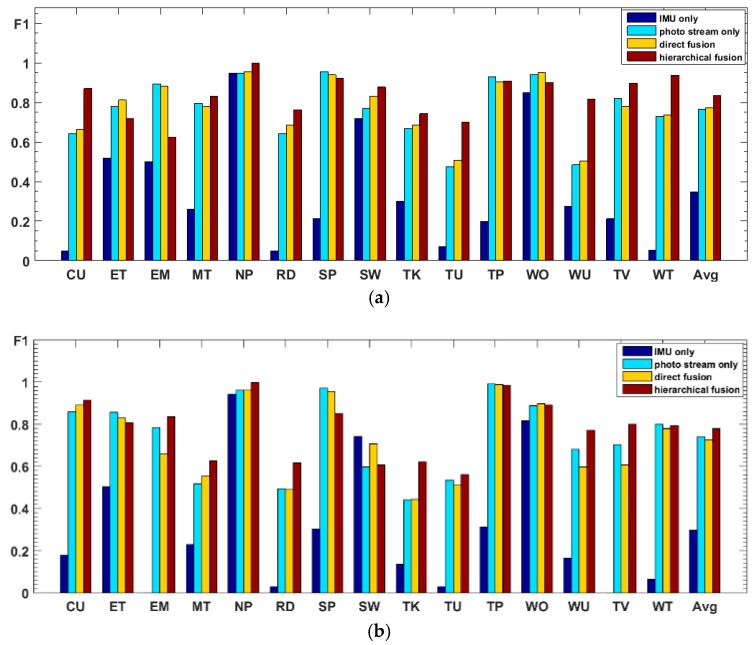
*F*_1_ accuracies of four methods shown as bar graphs for (**a**) W1 and (**b**) W2.

**Comparison and discussion:** In the direct fusion method proposed in [14], the LSTM (for motion sensor data classification) and the ConvNets (for video classification) operate in the same classification mode, i.e., both of them are used for classifying all of the 20 activities. Therefore, when the direct fusion method is applied to the eButton Dataset, the $LSTM^{x}$ (for IMU motion sensor classification) and the fine-tuned $VGG_{conv}$ (for photo stream classification) are both used to classify all 15 activities. When both $LSTM^{x}$ and $VGG_{conv}$ work in the same classification mode, the confusion matrices of the classification results of W1 and W2 are as shown in Figure 16 and Figure 17, respectively. The direct fusion architecture shown in Figure 11 is then used for fusing the classification results of $LSTM^{x}$ and $VGG_{conv}$, and the confusion matrices of the direct fusion results are as shown in Figure 18. The *F*_1_ accuracy of direct fusion is shown in Table 11. For comparison, the *F*_1_ accuracy of the IMU sensor data alone for all 15 activities, *F*_1_ accuracy of the photo stream alone for all 15 activities, *F*_1_ accuracy of direct fusion, and *F*_1_ accuracy of the proposed hierarchical deep fusion framework are all displayed in the same bar graph, as shown in Figure 19.

From the comparison results in Figure 19, it can be seen that, when the IMU data are used to classify all 15 activities, the IMU data only have good recognition results for some activities with obvious changes in motion state, such as “nap,” “sweeping,” and “walking outside.” For other activities, especially sedentary activities, the ability to distinguish is very poor, which makes the recognition results of IMU data very different from that of the photo stream. Therefore, when the recognition results of IMU data are directly fused with the results of the photo stream, the fusion results are not only not obviously improved but may even decrease (such as the recognition results of W2); i.e., the recognition results of IMU data have little or even a negative effect on the fusion process. In contrast, in the proposed hierarchical deep fusion framework, the IMU data are only used to distinguish the motion state so as to function in its best classification mode; thus, it plays a good role in promoting the fusion result. Compared with direct fusion, the fusion result of the proposed framework is a significant improvement, and the average accuracy can be increased by about 6%. Meanwhile, for the activities that are likely to occur in different motion states, such as “reading,” “talking,” “telephone use,” and “watching TV,” as they are classified into different groups with fewer candidate activities by IMU data, their recognition accuracy is substantially improved. In addition, some of the activities, such as “eating,” “walking outside,” the “entertainment” of W1, and the “sweeping” of W2, can be recognized more accurately by the direct fusion method than with the proposed hierarchical deep fusion method. The main reason is that the difference between the accuracies of the IMU data and photo stream for these activities is relatively small; thus, the results from these two kinds of sensors complement each other when directly fusing. In contrast, the proposed method uses a single sensor in each layer, which may counteract this complementary result when the single sensor misrecognizes an activity. Therefore, the direct fusion method is more suitable for applications with small sensor differences, whereas, when the sensor difference is large, the proposed method will be more competent.

#### 4.3.2. Results on the Multimodal Dataset

**Figure 20:**
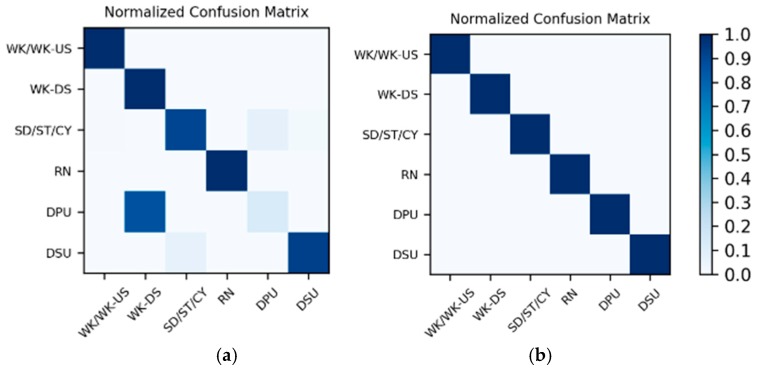
Confusion matrices for the two splits with (**a**) the lowest accuracy and (**b**) the highest accuracy on motion sensor data in the Multimodal Dataset.

**Results on motion sensor data:** The 200 sequences in the Multimodal Dataset are divided into 10 splits, and the training and testing are completed by leave-one-out cross-validation, which is equivalent to 10 groups of one-to-one corresponding training sets and test sets. As a result, the actual number of training and testing is 10, and there is a total of 10 $LSTM^{x}$ corresponding to each split. For each of the 10 test results, the average accuracy calculating 10 splits is shown in Table 12. Meanwhile, in the test results of the 10 splits, the confusion matrices corresponding to the two splits with the lowest accuracy and the highest accuracy are shown in Figure 20.

**Figure 21:**
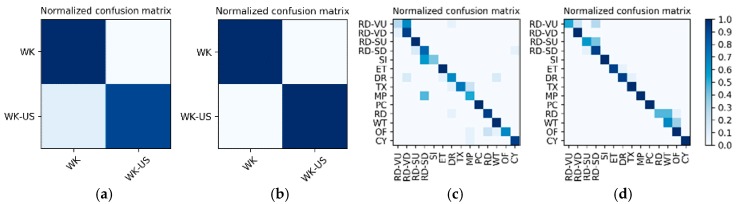
Lowest and highest accuracy confusion matrices for the VGG16-LSTM network corresponding to each group: (**a**,**b**) the WK/WK-US group; (**c**,**d**) the “SD/ST/CY group.

**Results on high-frame rate photo stream:** The photo streams are classified into the groups defined by ${ℳ}_{6}$ in Table 4, so the hybrid data in the Multimodal Dataset were also adjusted according to the group, and the fine-tuned VGG16-LSTM network shown in Figure 7 was established in each of the six groups to complete the training and testing. In addition, similar to the results on motion sensor data, the VGG16-LSTM network and its test results also needed to distinguish 10 splits. In all splits, the lowest and highest accuracy confusion matrices for the VGG16-LSTM network corresponding to each group were as shown in Figure 21. It should be noted that in the groups defined by ${ℳ}_{6}$ in Table 4, only ${C^{-1}}_{A↔ℳ}(ʺ\mathrm{WK}/\mathrm{WK-US}ʺ)$ and ${C^{-1}}_{A↔ℳ}(ʺ\mathrm{SD}/\mathrm{ST}/\mathrm{CY}ʺ)$ contain multiple activities, so only these two groups needed to train the VGG16-LSTM network. Thus, Figure 21 contains only the confusion matrices corresponding to these two groups. The average accuracy of the 10 splits for each activity in the group is shown in Table 13 and Table 14.

**Figure 22:**
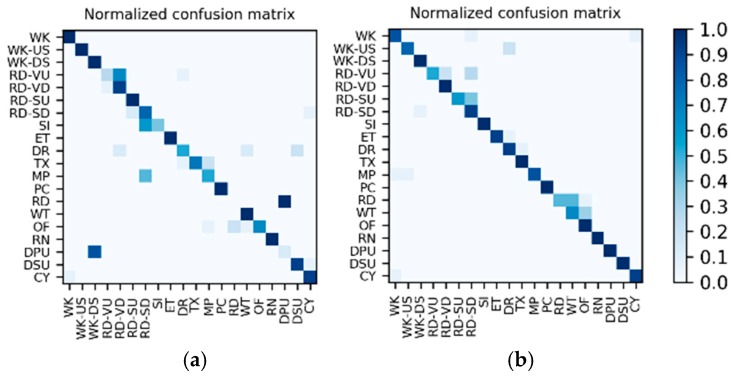
After hierarchical fusion, among the 10 splits, the confusion matrices with the (**a**) lowest accuracy and (**b**) highest accuracy.

**Results of the hierarchical combination:** After both the recognition results of motion sensor and the recognition results of photo stream are obtained, the hierarchical fusion results can be completed by referring to the hierarchical architecture shown in Figure 8. After fusion, among the 10 splits, the confusion matrices with the lowest and highest accuracy were as shown in Figure 22. The average accuracy of the 10 splits for each activity to be recognized is shown in Table 15.

**Figure 23:**
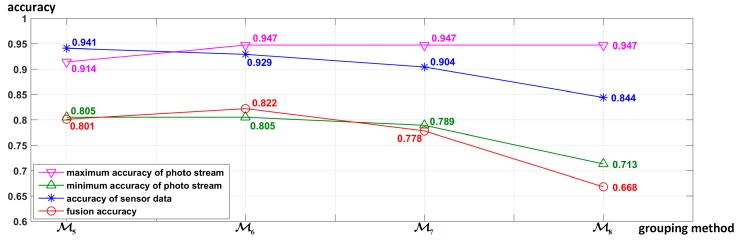
Accuracy comparison of different grouping methods shown in Table 4 used in the proposed hierarchical deep fusion framework.

**Influence of different grouping methods on fusion accuracy:** As different grouping methods will change the input data and classification mode of the deep neural network classifier (LSTM, CNN, or CNN-LSTM), the grouping method will also affect the recognition results of both the motion state layer and the activity layer, which in turn will lead to different fusion accuracies. In order to evaluate the influence of different grouping methods on the fusion accuracy, the four different grouping methods shown in Table 4 were used to perform the proposed hierarchical deep fusion framework. All of the accuracies are shown in Figure 23. To analyze how the accuracy of different layers influences the fusion results, Figure 23 shows both the accuracy of the motion sensor data (motion state layer) and that of the photo stream (activity layer) in each grouping method, wherein all accuracies are the average accuracy of 10 splits. Furthermore, for the photo stream, different grouping methods have different numbers of photo stream accuracies due to different numbers of groups. Therefore, to compare multiple photo stream accuracies with the fusion accuracy, only the maximum and minimum accuracies of each photo stream corresponding to different groups using each grouping method are shown in Figure 23.

Figure 23 reveals the following: (1) The number of groups is closely related to the accuracy of the grouping. In general, the fewer the groups, the more similar activities are merged, the greater the difference between the groups, and the higher the accuracy of grouping. (2) When the number of groups decreases, the number of activities in each group increases, which usually leads to a decrease in the difference among the activities within the group, thereby reducing the recognition accuracy of the photo stream. Therefore, the number of groups should not be too large or too small, and a compromise is needed. Overall, however, the accuracy of sensor data is more closely related to the fusion accuracy, i.e., the performance of the motion state layer is more important to the fusion result than the activity layer.

**Comparison and discussion:** In this part, we directly compare the results of the proposed hierarchical deep fusion framework with the fusion results given in [14] by using the multistream direct fusion method. As in the experimental results section in [14], only the average accuracy of each stream on 10 splits (as shown in Figure 11) and the average accuracy after direct fusion on 10 splits are given, we also list the accuracy of the corresponding items possible to complete the comparison of the algorithm results, as shown in Table 16.

Further, we compare the time consumption of the two algorithms. As the actual time consumed by the algorithm proposed in [14] is not reported, we can only rely on its algorithm flow to sum the time consumption estimation results of each key algorithm. In the online recognition process, as shown in Figure 11, the algorithm flow of the algorithm proposed in [14] mainly includes two optical flow field extraction calculations, three identical CovNets calculations, and four identical LSTM (denoted by $LSTM_{1}$) calculations. During the execution of the algorithm, the video and motion sensor data are not processed synchronously; specifically, for the video, each frame in the video is processed, while for the motion sensor data, the data within the time segment are processed. Therefore, if the calculation time of the optical flow field extraction of a single frame is defined as $t_{OF}$, the calculation time of a single $LSTM_{1}$ is $t_{L1}$, and the calculation time of a single CovNets is $t_{C1}$, the inferred range of total time consumption $t_{1}$ for the single-frame data in [14] is as follows:

(12) $$2\cdot t_{OF}+3\cdot t_{C1}\leq t_{1}\leq 4\cdot t_{L1}+2\cdot t_{OF}+3\cdot t_{C1}.$$

In the online recognition process of the proposed algorithm, the algorithm flow mainly includes one $LSTM^{x}$ calculation for the motion state grouping and one VGG16-LSTM calculation for the single-frame image recognition in its corresponding group. Similarly, because the two LSTMs ($LSTM^{x}$ and $LSTM_{j}^{I}$) and VGG-16 are also not processed synchronously, if the calculation time of $LSTM^{x}$ is $t_{L2}$, that of VGG16-LSTM is $t_{C-L}$, and that of VGG-16 CNN is $t_{C2}$, then the range of total time consumption $t_{2}$ for single-frame data in the proposed algorithm is as follows

(13) $$t_{C2}\leq t_{2}\leq t_{L2}+t_{C-L}.$$

$t_{C2}$, $t_{L2}$ and $t_{C-L}$ in Equation (13) can be directly measured on the experimental platform used in this paper (described in Section 4.2). The inferred values of $t_{L1}$ and $t_{C1}$ in Equation (12) can be measured by running the same $LSTM_{1}$ and CovNets (constructed according to the detailed network structure described in [14]) on our experimental platform. The measured values of the above calculation times are shown in Table 17. It should be noted that, in the measurement process, the input data frames of the two algorithms are exactly the same. For $LSTM_{1}$ and $LSTM^{x}$, the input data frame is a 3 s time segment, and for CovNets and VGG16-LSTM, the input frame is a single frame image scaled to 224 × 224 × 3. 

According to the specific real-time optical flow algorithm (TV-L^1^) used in [14], in Equation (12) is determined to be $t_{OF}\approx 12.2\;ms$ (with a resolution of 256 × 256 and 25 iterations) based on the measured results presented in [49,50], which are cited by [14]. Substituting $t_{OF}$ and the values of $t_{L1}$ and $t_{C1}$ in Table 17 into Equation (12), the inferred range of $t_{1}$ can be determined to be $53.527\;ms\leq t_{1}\leq 56.163\;ms$. Substituting the values of $t_{C2}$, $t_{L2}$, and $t_{C-L}$ in Table 17 into Equation (13), the range of $t_{2}$ can be determined to be $2.395\;ms\leq t_{2}\leq 4.762\;ms$. The frame rates corresponding to $t_{1}$ and $t_{2}$ are $18\leq fps_{1}\leq 19$ and $209\leq fps_{2}\leq 418$, respectively. Note that $t_{1}$ of the algorithm proposed in [14] is much longer than $t_{2}$ of the proposed algorithm because the optical flow calculation is very time-consuming.

As can be seen from the comparison in Table 16, when only the motion sensor data are used to complete the recognition of all 20 activities in the activity set, the recognition accuracy is also much lower than the video-based recognition accuracy (49.5% vs. 75%). Therefore, when the direct fusion framework shown in Figure 11 is used for fusion, the promotion of motion sensor-based results to video-based results is also limited. In contrast, by using the proposed hierarchical deep fusion framework, the number of candidate activities in the group is greatly reduced after the activities to be recognized are divided into different groups with the help of motion sensor data. As a result, the recognition accuracy of each activity is improved to some extent so that the overall average recognition accuracy after fusion is also improved. In addition, although the average recognition accuracy after hierarchical fusion is not much higher than that in [14] (82.2% vs. 80.5%), the proposed framework does not use a time-consuming optical flow field extraction algorithm to extract the optical flow field between adjacent frames. Meanwhile, in the process of online recognition, only two deep networks ($LSTM^{x}$ and VGG16-LSTM) are involved in the processing of motion sensor data and photo stream (the number of online deep networks is seven in the framework proposed in [14]), so the time complexity of the proposed hierarchical framework is much lower than that of the framework proposed in [14] (minimum frame rate of 209 vs. 18); thus, it is more suitable for practical applications.

## 5. Conclusions and Future Work

A deep-learning-based hierarchical fusion framework for egocentric activity recognition using a wearable hybrid sensor system is proposed in this paper. The proposed framework was applied to fuse egocentric motion sensor data and a photo stream to complete ADL recognition of the wearer on a self-developed hybrid wearable life logger (eButton). In the proposed framework, LSTM and CNN (or CNN-LSTM) networks are used in different layers to obtain the recognition results of motion sensor data and photo stream, respectively. The motion sensor data are used only to distinguish the motion states of all the activities and classify them according to their motion states, while the photo stream is used to further complete the specific activity recognition in the motion state groups. For the photo stream, based on the temporal correlation among the images determined by the record frame rate, we perform the activity recognition in the motion state groups by using the pre-trained CNN or CNN-LSTM, respectively. The experimental results show that the proposed hierarchical deep fusion framework can make the motion sensor data and the photo stream work in their most suitable classification mode, so as to effectively eliminate the negative influence of sensor differences on the fusion results. Further, compared to the existing direct fusion framework, the proposed hierarchical deep fusion framework increases the average accuracy by up to 6%. In addition, as time-consuming calculations such as the extraction of the optical flow field are avoided, the time complexity of the proposed framework is much lower than that of the existing direct fusion framework, making it more suitable for practical applications.

In the algorithm flow of the proposed framework, the accuracy of the grouping in the motion state layer is very important, which will directly affect the final recognition accuracy. Therefore, future work on the proposed framework will focus on further increasing the accuracy of the motion state grouping. Considering that the current motion state grouping is mainly done by LSTM on the motion sensor data, the accuracy of the motion state grouping can be improved in the following two ways: (1) From the network aspect, we can attempt to increase the depth of the LSTM or switch to a bidirectional LSTM to better describe the temporal characteristics of the motion sensor data. (2) From the data aspect, the fusion-based method can also be introduced to combine the motion sensor data and the photo stream in the motion state layer to overcome the limitations of a single motion sensor.

## Figures and Tables

**Table 1:** Number of time segments in the training and test sets.

Dataset		LY	SD	ST	WK	Total
training set	W1	593	607	602	576	2378
	W2	626	605	621	523	2375
test set	W1	178	1332	113	146	1769
	W2	199	1133	120	98	1550

**Table 2:** Number of images in the training and test sets.

Dataset		CU	ET	EM	MT	NP	RD	SP	SW	TK	TU	TP	WO	WU	TV	WT	Total
training set	W1	139	115	117	153	153	146	170	127	79	106	185	188	102	84	97	1961
	W2	119	149	105	120	107	84	112	123	80	95	106	97	113	101	108	1619
test set	W1	138	155	59	92	178	101	149	113	70	99	184	146	90	70	125	1769
	W2	95	159	87	95	197	91	91	120	42	98	79	98	95	94	109	1550

**Table 3:** The egocentric activities and their corresponding categories in Multimodal Dataset.

**Ambulation**	
1	walking (WK)
2	walking upstairs (WK-US)
3	walking downstairs (WK-DS)
4	riding elevator up (RD-VU)
5	riding elevator down (RD-VD)
6	riding escalator up (RD-SU)
7	riding escalator down (RD-SD)
8	sitting (SI)
**Daily Activities**	
9	eating (ET)
10	drinking (DR)
11	texting (TX)
12	making phone calls (MP)
**Office Work**	
13	working at PC (PC)
14	reading (RD)
15	writing sentences (WT)
16	organizing files (OF)
**Exercise**	
17	running (RN)
18	doing push-ups (DPU)
19	doing sit-ups (DSU)
20	cycling (CY)

**Table 4:** All grouping methods established during the adjustment of the grouping method when the proposed algorithm is applied to the Multimodal Dataset.

${ℳ}_{5}$	ℳ	{ʺWK/WK-US/WK-DS,ʺ ʺSD/ST/CY,ʺ ʺRN,ʺ ʺDPU,ʺ ʺDSUʺ}
	$C_{A↔ℳ}$	$C^{-1}(ʺ\mathrm{WK}/\mathrm{WK-US}/\mathrm{WK-DS}ʺ)=\{ʺ\mathrm{WK},ʺ\;ʺ\mathrm{WK-US},ʺ\;ʺ\mathrm{WK-DS}ʺ\}$, $\begin{array}{ll} C^{-1}(ʺ\mathrm{SD}/\mathrm{ST}/\mathrm{CY}ʺ)= & \{ʺ\mathrm{RD-VU},ʺ\;ʺ\mathrm{RD-VD},ʺ\;ʺ\mathrm{RD-SU},ʺ\;ʺ\mathrm{RD-SD},ʺ\;ʺ\mathrm{SI},ʺ\;ʺ\mathrm{ET},ʺ\;ʺ\mathrm{DR},ʺ\;ʺ\mathrm{TX},ʺ\;ʺ\mathrm{MP},ʺ\\ & ʺ\mathrm{PC},ʺ\;ʺ\mathrm{RD},ʺ\;ʺ\mathrm{WT},ʺ\;ʺ\mathrm{OF},ʺ\;ʺ\mathrm{CY}ʺ\}, \end{array}$ $C^{-1}(ʺ\mathrm{RN}ʺ)=\{ʺ\mathrm{RN}ʺ\}$, $C^{-1}(ʺ\mathrm{DPU}ʺ)=\{ʺ\mathrm{DPU}ʺ\}$, $C^{-1}(ʺ\mathrm{DSU}ʺ)=\{ʺ\mathrm{DSU}ʺ\}$.
${ℳ}_{6}$	ℳ	{ʺWK/WK-US,ʺ ʺWK-DS,ʺ ʺSD/ST/CY,ʺ ʺRN,ʺ ʺDPU,ʺ ʺDSUʺ}
	$C_{A↔ℳ}$	$C^{-1}(ʺ\mathrm{WK-DS}ʺ)=\{ʺ\mathrm{WK-DS}ʺ\}$, $\begin{array}{ll} C^{-1}(ʺ\mathrm{SD}/\mathrm{ST}/\mathrm{CY}ʺ)= & \{ʺ\mathrm{RD-VU},ʺ\;ʺ\mathrm{RD-VD},ʺ\;ʺ\mathrm{RD-SU},ʺ\;ʺ\mathrm{RD-SD},ʺ\;ʺ\mathrm{SI},ʺ\;ʺ\mathrm{ET},ʺ\;ʺ\mathrm{DR},ʺ\;ʺ\mathrm{TX},ʺ\;ʺ\mathrm{MP},ʺ\\ & ʺ\mathrm{PC},ʺ\;ʺ\mathrm{RD},ʺ\;ʺ\mathrm{WT},ʺ\;ʺ\mathrm{OF},ʺ\;ʺ\mathrm{CY}ʺ\}, \end{array}$ $C^{-1}(ʺ\mathrm{RN}ʺ)=\{ʺ\mathrm{RN}ʺ\}$, $C^{-1}(ʺ\mathrm{DPU}ʺ)=\{ʺ\mathrm{DPU}ʺ\}$, $C^{-1}(ʺ\mathrm{DSU}ʺ)=\{ʺ\mathrm{DSU}ʺ\}$.
${ℳ}_{7}$	ℳ	{ʺWK/WK-US,ʺ ʺWK-DS,ʺ ʺSD/STʺ ʺRN,ʺ ʺDPU,ʺ ʺDSU,ʺ ʺCYʺ}
	$C_{A↔ℳ}$	$C^{-1}(ʺ\mathrm{WK}/\mathrm{WK-US}ʺ)=\{ʺ\mathrm{WK},ʺ\;ʺ\mathrm{WK-US}ʺ\}$, $C^{-1}(ʺ\mathrm{WK-DS}ʺ)=\{ʺ\mathrm{WK-DS}ʺ\}$, $\begin{array}{ll} C^{-1}(ʺ\mathrm{SD}/\mathrm{ST}ʺ)= & \{ʺ\mathrm{RD-VU},ʺ\;ʺ\mathrm{RD-VD},ʺ\;ʺ\mathrm{RD-SU},ʺ\;ʺ\mathrm{RD-SD},ʺ\;ʺ\mathrm{SI},ʺ\;ʺ\mathrm{ET},ʺ\;ʺ\mathrm{DR},ʺ\;ʺ\mathrm{TX},ʺ\;ʺ\mathrm{MP},ʺ\;ʺ\mathrm{PC},ʺ\\ & ʺ\mathrm{RD},ʺ\;ʺ\mathrm{WT},ʺ\;ʺ\mathrm{OF}ʺ\}, \end{array}$ $C^{-1}(ʺ\mathrm{RN}ʺ)=\{ʺ\mathrm{RN}ʺ\}$, $C^{-1}(ʺ\mathrm{DPU}ʺ)=\{ʺ\mathrm{DPU}ʺ\}$, $C^{-1}(ʺ\mathrm{DSU}ʺ)=\{ʺ\mathrm{DSU}ʺ\}$, $C^{-1}(ʺ\mathrm{CY}ʺ)=\{ʺ\mathrm{CY}ʺ\}$.
${ℳ}_{8}$	ℳ	{ʺWK/WK-US,ʺ ʺWK-DS,ʺ ʺSD,ʺ ʺST,ʺ ʺRN,ʺ ʺDPU,ʺ ʺDSU,ʺ ʺCYʺ}
	$C_{A↔ℳ}$	$C^{-1}(ʺ\mathrm{WK}/\mathrm{WK-US}ʺ)=\{ʺ\mathrm{WK},ʺ\;ʺ\mathrm{WK-US}ʺ\}$, $C^{-1}(ʺ\mathrm{WK-DS}ʺ)=\{ʺ\mathrm{WK-DS}ʺ\}$, $C^{-1}(ʺ\mathrm{SD}ʺ)=\{ʺ\mathrm{SI},ʺ\;ʺ\mathrm{ET},ʺ\;ʺ\mathrm{DR},ʺ\;ʺ\mathrm{TX},ʺ\;ʺ\mathrm{MP},ʺ\;ʺ\mathrm{PC},ʺ\;ʺ\mathrm{RD},ʺ\;ʺ\mathrm{WT},ʺ\;ʺ\mathrm{OF}ʺ\}$, $C^{-1}(ʺ\mathrm{ST}ʺ)=\{ʺ\mathrm{RD-VU},ʺ\;ʺ\mathrm{RD-VD},ʺ\;ʺ\mathrm{RD-SU},ʺ\;ʺ\mathrm{RD-SD},ʺ\;ʺ\mathrm{ET},ʺ\;ʺ\mathrm{DR},ʺ\;ʺ\mathrm{TX},ʺ\;ʺ\mathrm{MP},ʺ\;ʺ\mathrm{RD},ʺ\;ʺ\mathrm{OF}ʺ\},$ $C^{-1}(ʺ\mathrm{RN}ʺ)=\{ʺ\mathrm{RN}ʺ\}$, $C^{-1}(ʺ\mathrm{DPU}ʺ)=\{ʺ\mathrm{DPU}ʺ\}$, $C^{-1}(ʺ\mathrm{DSU}ʺ)=\{ʺ\mathrm{DSU}ʺ\}$, $C^{-1}(ʺ\mathrm{CY}ʺ)=\{ʺ\mathrm{CY}ʺ\}$.

**Table 5:** *F*_1_ accuracy of the classification results on the IMU sensor data.

	LY	SD	ST	WK	Avg.
**W1**	1.0000	0.8482	0.9000	0.9388	0.9217
**W2**	1.0000	0.9212	0.7168	0.7945	0.8581

**Table 6:** *F*_1_ accuracy of the lying group (LY).

LY	NP	TU	Avg.
**W1**	0.9899	0.9794	0.9846
**W2**	0.9493	0.8939	0.9216

**Table 7:** *F*_1_ accuracy of the sedentary group (SD).

SD	CU	ET	EM	MT	RD	SP	TK	TU	TP	TV	WT	Avg.
**W1**	0.8814	0.9152	0.9212	0.8471	0.8323	0.9727	0.7475	0.7373	0.9375	0.9080	0.9422	0.8766
**W2**	0.9000	0.8765	0.9483	0.4154	0.5357	0.9732	0.4571	0.5616	0.9946	0.8099	0.7500	0.7475

**Table 8:** *F*_1_ accuracy of the standing group (ST).

ST	ET	EM	RD	SP	SW	TK	TU	TP	WU	Avg.
**W1**	0.8805	0.9202	0.8715	0.9630	0.8988	0.8293	0.8402	0.9202	0.8770	0.8890
**W2**	0.9067	0.7843	0.5673	0.8916	0.6667	0.4632	0.7344	0.9892	0.9071	0.7678

**Table 9:** *F*_1_ accuracy of the walking group (WK).

WK	SP	WO	Avg.
**W1**	0.9780	0.9796	0.9788
**W2**	0.9933	0.9932	0.9932

**Table 10:** *F*_1_ accuracy of the hierarchical fusion results.

	CU	ET	EM	MT	NP	RD	SP	SW	TK	TU	TP	WP	WU	TV	WT	Avg.
**W1**	0.8701	0.7188	0.6250	0.8304	0.9975	0.7630	0.9215	0.8772	0.7423	0.6992	0.9091	0.9000	0.8166	0.8966	0.9375	0.8336
**W2**	0.9158	0.8057	0.8364	0.6265	0.9972	0.6170	0.8504	0.6061	0.6218	0.5623	0.9865	0.8915	0.7712	0.8000	0.7939	0.7788

**Table 11:** *F*_1_ accuracy of the direct fusion results.

	CU	ET	EM	MT	NP	RD	SP	SW	TK	TU	TP	WP	WU	TV	WT	Avg.
**W1**	0.6626	0.8111	0.8810	0.7795	0.9540	0.6857	0.9392	0.8322	0.6863	0.5086	0.9036	0.9510	0.5038	0.7816	0.7358	0.7744
**W2**	0.8912	0.8308	0.6598	0.5537	0.9622	0.4895	0.9565	0.7064	0.4444	0.5130	0.9892	0.8985	0.5970	0.6081	0.7785	0.7253

**Table 12:** Average *F*_1_ accuracy calculating 10 splits on motion sensor data in the Multimodal Dataset.

	WK/WK-US	WK-DS	SD/ST/CY	RN	DPU	DSU	Avg.
**Average Accuracy on 10 Splits**	0.9082	0.8974	0.9825	0.9322	0.9000	0.9564	0.9294

**Table 13:** Average *F*_1_ accuracy of the WK/WK-US group.

WK/WK-US	WK	WK-US	Avg.
**Average accuracy on 10 splits**	0.948	0.945	0.947

**Table 14:** Average *F*_1_ accuracy of the SD/ST/CY group.

SD/ST/CY	RD-VU	RD-VD	RD-SU	RD-SD	SI	ET	DR	TX	MP	PC	RD	WT	OF	CY	Avg.
**Average accuracy on 10 splits**	0.631	0.705	0.924	0.842	0.880	0.864	0.713	0.843	0.599	0.978	0.835	0.876	0.714	0.866	0.805

**Table 15:** Average *F*_1_ accuracy of the 10 splits for each activity to be recognized.

	WK	WK-US	WK-DS	RD-VU	RD-VD	RD-SU	RD-SD	SI	ET	DR	TX	MP	PC	RD	WT	OF	RN	DPU	DSU	CY	Avg.
**Average precision on 10 splits**	0.881	0.832	0.897	0.631	0.698	0.915	0.837	0.880	0.851	0.687	0.843	0.551	0.978	0.747	0.875	0.721	0.932	0.900	0.956	0.823	0.822

**Table 16:** The direct fusion proposed in [14] and the proposed hierarchical fusion on the Multimodal Dataset.

	Direct Fusion Proposed in [14]		Hierarchical Fusion Proposed in This Paper
	Average Pooling	Maximum Pooling	
**Video/Photo Stream**	**68.5**% on 20 activities	**75%** on 20 activities	**94.7%** on 2 activities in group ${C^{-1}}_{A↔ℳ}(ʺ\mathrm{WK}/\mathrm{WK-US}ʺ)$
			**80.5%** on 14 activities in group ${C^{-1}}_{A↔ℳ}(ʺ\mathrm{SD}/\mathrm{ST}/\mathrm{CY}ʺ)$
**Sensor Data**	**49.5%** on 20 activities		**92.9%** on 6 motion groups
**Fusion**	**76.5%**	**80.5%**	**82.2%**

**Table 17:** Measured calculation times in Equations (12) and (13).

$t_{C1}$ (ms)	$t_{L1}$ (ms)	$t_{C2}$ (ms)	$t_{L2}$ (ms)	$t_{C-L}$ (ms)
9.709	0.659	2.395	0.659	4.103
